# Linking emotion regulation strategies to employee motivation: The mediating role of guanxi harmony in the Chinese context

**DOI:** 10.3389/fpsyg.2022.837144

**Published:** 2022-07-27

**Authors:** Wenpei Zhang, Shanshan Guo, Jiashu Liu, Ying He, Mengmeng Song, Lirong Chen

**Affiliations:** ^1^Department of Business Administration, School of Business, Anhui University of Technology, Maanshan, China; ^2^School of Management, Xi’an Jiaotong University, Xi’an, China; ^3^Department of Psychology, Suzhou University of Science and Technology, Suzhou, China

**Keywords:** employee motivation, guanxi harmony, leadership, reappraisal, suppression

## Abstract

This study examined the mediating role of guanxi harmony, a concept of interpersonal relationships specific to the Chinese context, between leaders’ emotion regulation strategies and employee motivation. Data were drawn from 489 on-the-job MBA students with enough management experience from thirteen different types of cities in China. The study tested the model using hierarchical regression. The results showed that the reappraisal strategy was positively related to employee motivation and the suppression strategy was negatively related to employee motivation in the Chinese context. Guanxi harmony played a partially mediating role between reappraisal and employee motivation, and played a fully mediating role between suppression and employee motivation. These findings suggest that in the Chinese context, guanxi harmony between leaders and employees has a positive effect on employee motivation, and when leaders apply appropriate emotion regulation strategies, they can achieve guanxi harmony and promote employee motivation.

## Introduction

Employee motivation is one of the essential qualities of effective leadership (e.g., transformational leadership, Hamstra et al., 2014; entrepreneurial leadership, Mehmood et al., 2021). Employee motivation can help employees not only mobilize their efforts, transcend their interests, and work together for organizational goals, but it can also provide them with more security, reduce the stress and pressure brought on by unpredictable internal and external situations, assist them in coping with setbacks, and improve their resilience (Han et al., 2016). Therefore, employee motivation is increasingly essential in complex organizational situations. Leaders with motivational qualities can portray a clear and vivid vision, inspire their higher-level needs through communication with employees, and pursue more worthy goals. Specifically, when leaders successfully motivate employees, employees can emphasize the interests of the organization. Specifically, when leaders successfully motivate employees, employees may stress the organization’s interests. Employees tend to have more positive emotions, higher work efficiency, and workplace innovation to help the organization reach qualifying goals and maintain organizational harmony. Employee job autonomy should also be improved, so that employees can be more proactive and better cope with setbacks and negative emotions (Abu Orabi, 2016).

Most existing research has focused on the consequences of employee motivation, with few studies examining its antecedents. The antecedents of employee motivation have previously been classified into two aspects: the organizational factors, such as characteristics of the job (e.g., job autonomy; Engert and Baumgartner, 2016) and organizational context (e.g., organizational culture; Engert and Baumgartner, 2016); and the individual factors, such as personalities of leaders and emotions (Ghani et al., 2016; Alam, 2020). Emotions, as a link between people, play a critical role in the interaction process of employee motivation between the leaders and their subordinate employees (Gooty et al., 2010). Although the previous studies have emphasized the importance of emotions (Kaplan et al., 2014), there are limited studies on how to use emotions to increase employee motivation.

The leader’s emotions have a direct impact on the emotional state of the entire team, as well as the behaviors and work efficiency of employees (Bhullar, 2012). Ideal leadership emotions can inspire ideal employee emotions, and leaders can positively motivate employees through the interaction between leaders and employees, positively affecting employees’ work behaviors and their work effectiveness (Kaplan et al., 2014; Li et al., 2020). When leaders experience negative emotions due to various challenges, stress or adverse events (Aycan and Shelia, 2018), they may show their feelings through facial expressions, tone of voice and body movements, which are then perceived by employees (Li et al., 2020), affecting employees’ emotions or motivation, and impairing employee and organizational performance (Wang et al., 2017). In this situation, leaders need to use emotion regulation strategies to manage their own and their employees’ emotions in order to mitigate the harmful impact of negative emotions on employee productivity and organizational effectiveness (Ayoko and Callan, 2010; Li et al., 2020). Usually, negative emotions will prompt leaders to apply emotion regulation strategies to express survival-adapted behaviors, with reappraisal and suppression being the two most common types of emotion regulation strategies applied by individuals (Dixon-Gordon et al., 2015). Reappraisal reduces or transforms the individual’s negative emotions by reappraising adverse events. In contrast, suppression lowers the impact of negative emotions on the individual’s psychology and behavior by preventing negative emotions from being expressed. Eventually, individuals who applied appropriate emotion regulation strategies could reduce the impairment to interpersonal interaction or the negative emotional infection they spread to others due to the inappropriate expression of negative emotions (Giombini, 2015).

Culture may influence the usage of emotion regulation strategies of leaders. There are significant cultural differences in the usage of emotion regulation strategies between Western cultures (e.g., the United States, Canada, and Australia) and Eastern cultures (e.g., China, Japan, and Korea; Kitayama and Uskul, 2011). Individuals cultures with individualistic value orientation in Western pursue independence and autonomy, and express their emotions more frequently. Suppression is considered to lead to more potentially adverse outcomes (e.g., anxiety, depression) due to its characteristics of suppressing emotions, which do not truly relieve negative emotions. Therefore, suppression is not regarded as an optimal emotion regulation strategy in Western cultures (Le and Impett, 2013). However, individuals with collectivist value orientation in Eastern cultures are more likely to apply the strategy of suppression. They believe that a harmonious collective requires tolerance and coordination with each other, so they often control their emotional expression to pursue the collective goal of harmonious relationships to achieve interpersonal harmony and collective goals (Wei et al., 2013). Although Eastern leaders prefer to use suppression, it remains to be verified whether this kind of emotion regulation strategy is conducive to transformational leadership and whether it can effectively improve employee motivations affected by negative emotions.

Furthermore, the mechanisms by which emotion regulation affects employee motivation may differ from cultures (Kitayama and Uskul, 2011). In Western cultures, business activities are primarily institutional or organizational that leaders influence employees’ work attitudes and behaviors based on rules or laws (Dasborough et al., 2009). However, in Eastern cultures, harmonious interpersonal relationships and the establishment of interpersonal and emotional relationships among individuals are emphasized. Business activities in Eastern cultures rely on interpersonal interaction, using a more “humanistic” rather than “legal” approach (Wang et al., 2008; Wu et al., 2020). Especially in China, guanxi, a Chinese idea of interpersonal relationships, has a profound impact on Chinese economic activity in every way (Chen et al., 2013). When disagreements emerge, this “humanistic” style of interpersonal interaction would be an ideal resolution strategy to handle them because leaders can inspire or get enough support from their team members to accomplish team and organizational goals if they have guanxi harmony with them (Walumbwa et al., 2005). The guanxi harmony requires individuals to have high-level emotion regulation ability to effectively understand and manage their own and others’ emotions, thereby maintaining good relationships with others. Thus, in Eastern cultures, interpersonal relationships may play an important intermediary role in the mechanism of emotion regulation affecting employee motivation.

In sum, the present study will explore the impact of different emotion regulation strategies of leaders on employee motivation in the Chinese context and clarify whether and how interpersonal relationships can play an important mediating role between emotion regulation and employee motivation.

## Theoretical background and hypotheses

### Emotion regulation strategies and employee motivation

The Emotion As Social Information (EASI) model can provide a theoretical basis for how leaders influence employee motivation through emotions (Van Kleef, 2009). Based on the social function of emotions and the information processing system of decision making, the model categorizes the emotional information processing of individuals toward others in two ways: emotional response and inferential processing. Emotional response refers to the emotional effect that the expresser directly stimulates the observer’s emotional state. The EASI model has been supported and applied in leadership studies (Côté, 2014). Based on the EASI model, the mechanism of the effect of emotion regulation strategies on employee motivation can be explained by way of emotional response. When encountering setbacks, the positive emotions of the leader will impact the employees, giving them a sense of security and boosting their confidence in handling challenges. This will lead to more positive emotions in the team members, which will motivate the employees. Accordingly, the leader’s negative emotions will damage the employees’ motivation by emotional infection and lowering their confidence (Visser et al., 2013). When negative emotions appear, leaders may apply different emotional regulation strategies, such as suppression and reappraisal, to reduce or change the current negative emotions, so that leaders can weaken or eliminate the negative impact of emotions on ongoing work (Hülsheger et al., 2014).

Emotion regulation strategies can reduce the individual’s perception and experience of failure, and promote improving and recovering the individual’s mental state. Previous study showed that when leaders and employees regulate their emotions, they can significantly improve leader-member exchange and promote work effectiveness (Richards and Hackett, 2012). Gross (1998) divided emotion regulation strategies into two types: reappraisal and suppression (Gross, 1998). Reappraisal refers to the process by which individuals change their understanding of a situation to change their emotional response to reduce negative emotions or to transform negative emotions into positive emotions. Suppression refers to the process by which individuals suppress their emotional response to reduce the adverse effects of negative emotions. Evidence from research conducted in Western countries suggested that the reappraisal emphasized the individual’s reconsideration of the current event and, therefore, cognitively changed the understanding and the attitude of the emotional events, altering the individual’s emotional response accordingly (Mohammed et al., 2021). Successful reappraisal can reduce negative emotions significantly and can even transform negative emotions into positive emotions (Richards and Gross, 2000; Robinson et al., 2021). Therefore, it is more likely to motivate employees intrinsically to perform their current job and achieve employee motivation. In contrast, the component of suppression regarding the suppression of natural emotional experiences leads individuals to generate a series of stress responses in individuals. Suppressing negative emotions that have existed and been experienced can lead to the blockage of normal outgoing pathways; thus, it could aggravate the negative emotional experiences, increase emotional pressure, produce emotional exhaustion, and cause resource imbalance and burnout (Schaubroeck and Jones, 2000; Ruan et al., 2020). Therefore, a suppression strategy may diminish leadership effectiveness (Dasborough, 2006).

Different cultures may influence the use of emotion regulation strategies. Western cultures consider suppression as a negative emotion regulation strategy and do not promote its use (Butler et al., 2007). Eastern cultures with collectivist values, however, tend to use suppression strategy more frequently (Kitayama and Uskul, 2011). They consider suppression as a strategy that facilitates harmonious collective goals by suppressing the expression of negative emotions, with the underlying concept of humility and consideration for others, to achieve a harmonious human-computer relationship satisfaction in the group (Le and Impett, 2013). Usually, positive emotions are often required for leaders to influence and motivate employees (Wijewardena et al., 2017), whereas suppression is more likely to only suppress and reduce negative emotions without increasing the positive emotions required for motivation. So although suppression has many benefits and has been even actively used in Eastern cultures (Butler et al., 2007), it may not be an effective strategy to promote employee motivation. On the other hand, reappraisal could convert negative emotions into positive emotions. Reappraisal refers to interventions before the complete process of emotional response and can be effective in changing the subsequent path of emotional evolution; that is, reappraisal can change the understanding of emotional events, avoiding negative emotions due to subconscious reactions and shifting negative emotions to positive emotional expressions. Thus, reappraisal is an adaptive emotion regulation strategy that adapts to the demands of the environment (Brewer et al., 2016), which is more likely to be an effective strategy to promote employee motivation. So we hypothesized that:

*H1: Reappraisal is positively related to employee motivation.*

*H2: Suppression is negatively related to employee motivation.*

### The mediating role of guanxi

Human relations theory emphasized the important role of interpersonal relationships in HRM (Methot et al., 2018). Bono and Ilies (2006) found that harmonious interpersonal relationships with superiors and subordinates could facilitate communication and interaction, allowing employees to freely express their true thoughts; Thiel et al. (2015) found that good interpersonal relationships could improve employees’ moods, promote changes in their positive emotions, and relieve work stress (Thiel et al., 2015); Barba-Sánchez and Molina Ramírez also found the key role of entrepreneurs’ social relationships in community groups as well as in economic activities (Barba-Sánchez and Molina Ramírez, 2015; Molina-Ramírez and Barba-Sánchez, 2021). The Chinese culture has both the collectivist spirit of Eastern culture and a unique cultural characteristic based on this culture – “guanxi” (Chen and Chen, 2009). Guanxi is the concept that uses networks of interpersonal connections to secure interests in personal and organizational relationships, and is important to the interpersonal and inter-organizational dynamics of Chinese society (Luo et al., 2016). The practice of guanxi has its roots in Confucianism, which nurtures a wide range of collectivist cultures, which is manifested in the importance of interpersonal networks. In Chinese society, guanxi is so pervasive that it is involved in all aspects of social and organizational life (Park and Luo, 2001; Li et al., 2019); also, it is the lifeblood of interpersonal and business behaviors in human resource management (Chen et al., 2013). In Chinese culture, business tends to be driven more by interpersonal interactions than by corporate systems, especially in organizations where employees place more value on interpersonal relationships and have a greater desire to integrate into the collective and communicate with others. To realize employee motivation needs to stimulate employees’ intrinsic motivation, which often requires employees’ internal recognition of the leader and the content of motivation; that is, leaders need to obtain employees’ support when motivating employees (Cuadrado et al., 2014). Guanxi harmony, the important humanistic approach in business activities, is crucial in obtaining employees’ internal support and inducing their internal recognition (Lam et al., 2015), so we need to consider the important role of guanxi harmony in the relationship between emotion regulation and employee motivation.

Reappraisal has significant positive implications in the face of conflict or adversity in both Western and Eastern cultures (Hua et al., 2015; Brewer et al., 2016). Reappraisal can help leaders understand or rationalize negative or challenging events in a more positive way when experiencing negative emotions such as frustration and disgust (Liu et al., 2021). Previous studies showed that reappraisal can reduce the experience and appropriate behavioral expression activated by negative emotions, without causing a loss of the individual’s limited cognitive resources and is less likely to trigger physiological reactions in employees. By weakening the individual’s negative emotional experience and increasing the positive emotional experience, the individual could have a more positive attitude toward the events, and could positively influence the rest of the individuals of the collective, thereby achieving a positive perception of the current situation for the entire collective (Goldin et al., 2008). This change from negative to positive attitudes is different from the suppression strategy that it is an internal cognitive change, that is, individuals will be more likely to accept the current situation so that the negative emotions will be weakened, or even individuals could be aware of the positive aspect of the current event so that the positive emotions will be increased (Brewer et al., 2016). Therefore, reappraisal could facilitate the resolution of interpersonal friction in the current situation. When applying reappraisal strategy, employees will try to rationalize more negative emotions and to understand the leader’s demands and reactions, and once the employees reach the enough rationalization of the negative emotions, they will generate fewer negative emotions and reduce internal emotional inconsistency, decreasing the depletion of psychological resources and the level of emotional exhaustion. In addition, such cognitive changes help employees focus more on self-goals and less on others’ evaluations, reduce frictional conflicts among team members, make employees to have more positive emotions and higher life satisfaction, and thus enhance psychological identification among team members which contributes to the deeper connections among interpersonal relationships (Hu et al., 2014). So psychological identification generated by reappraisal is likely to promote interpersonal harmony and employee motivation. Thus, we proposed the hypothesis that:

*H3: The positive relationship between reappraisal and employee motivation will be mediated by guanxi harmony.*

Different from reappraisal, East-West differences suggest that culture can have a significant impact on the use of suppression strategy. As discussed earlier, for Western individuals, suppression is often perceived as a maladaptive emotion regulation strategy (Brewer et al., 2016), while for Eastern individuals, suppression is not regarded as a negative emotion regulation strategy, but this strategy may even be perceived as contributing to interpersonal harmony and be expected to be applied to reduce the negative effect of negative emotions on the collective (Wei et al., 2013). A comparative study showed that the correlation between suppression and negative emotions in the Western cultural context is stronger than that in the Eastern cultural context (Hu et al., 2014). This suggested that the suppression strategy could bring some positive effects and was recognized to a certain extent in Eastern cultures.

In consideration of the complex effects of suppression in Eastern cultures, we need to explore its specific role in human resource management. Because if suppression can be potentially detrimental to leadership and we still use it in accordance with cultural habits, it can undermine leadership effectiveness. In Eastern cultures, the core of the suppression strategy is the repression of negative emotions, which can reduce negative emotions to some extent by suppressing negative emotional experiences and expressions, and temporarily restoring emotions to ensure the advancement of work (Soto et al., 2011). That is, compared to Western cultures, Chinese people are more able to benefit from the suppression strategy in terms of emotions; however, this emotional gain comes at the cost of more cognitive resources (Yuan et al., 2015), which can be potentially damaging to interpersonal relationships (Ferguson, 2012; Nam et al., 2018). According to resource conservation theory (COR, Hobfoll, 1989), the cognitive resources available to individuals are limited, there is competition for resources between different matters, and individuals will tend to allocate fewer resources to other matters in order to conserve resources for the matters they concern (Hobfoll et al., 2018). While the suppression strategy does not completely attenuate their stressful experiences, it could deplete limited individual resources in the process of suppressing the individuals’ real emotions, generate resource-protective behaviors such as decreased-proactive behaviors, and thus tend to reduce the resources which should be allocated to interpersonal relationships. And consequently, the interpersonal connections will be impaired, that is, it is impossible to create a deep trust relationship between individuals based on a sense of security (Ito and Brotheridge, 2003). Also, this stressful and negative emotional experience will further make individuals to apply expression depression strategy, which is detrimental to communication and exchange among leaders, colleagues, and subordinates, leading to interpersonal conflict in the long run.

The importance of guanxi in the Chinese business environment is well recognized that guanxi not only has an important impact on business affairs, but also on cooperative interactions (Cai et al., 2016), cognition and emotions of business members (Zhou et al., 2020). This means that guanxi could positively influence cooperation and altruistic behaviors among employees by affecting attitudes toward others and trust among people, facilitating resource exchange, strengthening favor exchange, solidifying status acquisition, and cognition and emotions (Bian, 1997). If the guanxi between leaders and employees is deeply damaged, the attitudes and trust of employees toward leaders will correspondingly be impaired. The influence of leaders on employees will tend to remain at the stage of institutional and authoritative obedience, instead of being able to stimulate employees’ internal identity, thus making it difficult for leaders to have the desired influence and emotional motivation on employees. So we proposed the hypothesis that:

*H4: The negative relationship between suppression and employee motivation will be mediated by guanxi harmony.*

**FIGURE 1:**
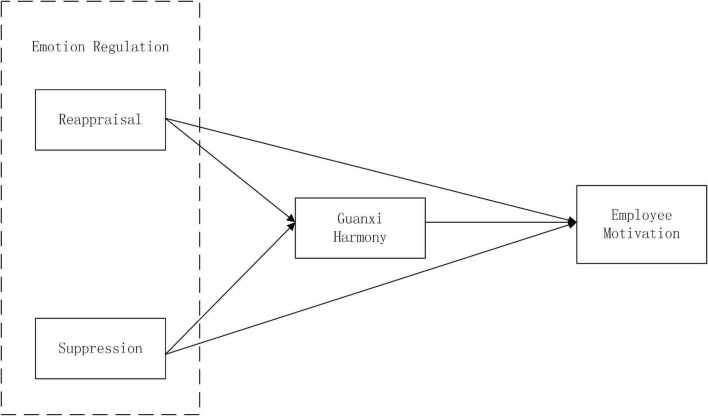
Theoretical model.

The research theoretical model is depicted in Figure 1.

## Materials and methods

### Participants

We recruited participants through posters or online advertisements from three universities in East China. Followed by previous studies (Ding et al., 2017). We had recruited 500 on-the-job Master of Business Administration students who had enough practical and management experience to participate in the present study. The participants came from thirteen various cities in China (i.e., first-tier cities such as Beijing and Shanghai, second-tier cities such as Changzhou, and third-tier cities such as Wuhu). After excluding the subjects whose answering time is too fast (less than 30 s) and/or with missing items, we collected 489 effective samples, and the questionnaire recovery rate is 97.8%. Table 1 shows the demographic description of the sample.

**TABLE 1:** Demographic information of sample.

Variables	Measurements	N (%)
Gender	Male	222 (45.4)
	Female	267 (54.6)
Age (years)	< 30	105 (21.4)
	31–35	331 (67.7)
	36–40	36 (7.3)
	> 40	17 (3.4)
Income (RMB/year)	< 50,000	130 (26.6)
	50,001–100,000	165 (33.7)
	100,001–150,000	132 (27.0)
	150,001–200,000	41 (8.4)
	> 200,000	21 (4.3)

### Measures

All items of measurement were adapted from prior validated scales, but some items were slightly changed to fit the current research context. Since we conducted the survey in China, we first translated the questionnaire from English to Chinese by two researchers from different backgrounds (i.e., management and psychology), following the standard forward-backward procedures (Van de Vijver and Leung, 2021). Then, a professional translator unfamiliar with the study was invited to translate the Chinese version back into English. No significant semantic differences were found between the translated questionnaire and the original English version.

#### Emotion regulation strategies

We used of Emotion Regulation Questionnaire (ERQ; Gross, 1998) to measure the emotion regulation strategies of leaders. This questionnaire concluded ten items which were used to measure the individual’s use of emotion regulation strategies. The questionnaire contained two dimensions which represented two types of strategies. The dimension of reappraisal reflected that individuals regulated their emotions by changing their cognition, concluding six items such as “When I want to feel less negative emotion, I change the way I am thinking about the situation.” The dimension of suppression reflected that individuals regulated their emotions by suppressing their emotions, concluding four items such as “I control my emotions by not expressing them.” All items were measured on a Likert 5-point scale, ranging from “Strongly disagree” to “Strongly agree.”

#### Guanxi harmony

We used the dimension of guanxi harmony of the Swift Guanxi Questionnaire to measure the status of guanxi harmony between the leaders and employees (Sun et al., 2019). This dimension concluded three items which were used to measure the degree of harmony of one’s guanxi, such as “I maintain harmony with each other at work.” All items were measured on a Likert 5-point scale, ranging from “Not at all” to “Frequently.”

#### Employee motivation

We used the dimension of inspiration motivation of the Multifactor Leadership Questionnaire (MLQ; Antonakis et al., 2003) to measure the employee motivation of the leaders. This questionnaire concluded four items which could help us to measure the capability of employee motivation from the leader’s self-assessment, such as “Articulates a compelling vision of the future.” All items were measured on a Likert 5-point scale, ranging from “Not at all” to “Frequently.”

#### Control variables

Based on previous research, we considered some demographic factors that may potentially impact the model as control variables to be included in the model calculation. For example, female leaders exhibit higher employee motivation than male leaders in transformational leadership style (Eagly et al., 2003). Age is positively related to transformational leadership, where employee motivation is measured as an important component of transformational leadership (Naber and Moffett, 2017). Although there was no direct evidence of the correlation between job-related variables such as income and position and employee motivation, we considered income as a control variable due to its significant correlation with mood and leadership style (Magaard et al., 2017). Therefore, we controlled for demographic variables such as gender, age, and income in the research model. These control variables were coded as ordinal categorical variables, in which: for gender, “1” = male, “2” = female; for age, “1” = 30 years and below, “2” = 31–35 years, “3” = 36–40 years, “4” = over 40 years; for income per year, “1” = RMB 50,000 and below, “2” = RMB 50,001–100,000, “3” = RMB 100,001–150,000, “4” = RMB 150,001–200,000, “5” = RMB 200,000 and above.

## Analysis and results

### Common method bias

Considering that we collected the data of our variables from a single source at a similar time, they may be affected by common method bias. Therefore, we used two methods to test for common method bias in this study. We first followed Harman’s single-factor test utilizing an exploratory factor analysis *via* SPSS 24.0. The results yielded four factors with eigenvalues greater than 1.0, and the first factor merely explained 31.082% of the variance (KMO = 0.857, χ2 = 3787.042, *p* < 0.001), which did not exceed the cut-off value of 50%. Besides, we used the common latent factor method to determine common method bias as it is found to be a better and reliable method (Podsakoff et al., 2003). We compared the fit values of the four-factor model with the model that included a common latent factor by Mplus 7.0. The results in Table 2 showed that the goodness of fit of the model with the common method latent factor did not improve significantly compared to the four-factor model (CFI and TLI increased by less than 0.1 and RMSEA decreased by no more than 0.05). This indicated that there is no serious common method bias in this study.

**TABLE 2:** Model fit values.

Model	x^2^	df	x^2^/df	CFI	TLI	SRMR	RMSEA
Four-factor model (reappraisal, suppression guanxi harmony, employee motivation)	281.681	113	2.492	0.955	0.945	0.046	0.055
Model with a common latent factor	193.928	97	1.999	0.974	0.963	0.034	0.045

χ^2^, Chi-square; df, degree of freedom; CFI, comparative fit index; TLI, Tucker-Lewis index; RMSEA, root mean square error of approximation; SRMR, standardized root mean square residual.

### Measurement model

To assess the measurement model of this study, we conducted reliability and validity tests. First, we used the constructs’ Cronbach’s α, composite reliability (CR), and average variance extracted (AVE) to assess the reliability and convergent validity. As shown in Table 3, the Cronbach’s α of all the constructs was significantly higher than the recommended value of 0.7 (Fornell and Larcker, 1981), which indicated that the measurement model of this study showed good reliability. The factor loadings of all items were higher than the threshold of 0.5 (Hair et al., 2010), and the CR ranged from 0.803 to 0.887, which was higher than the recommended threshold of 0.7, and the values of AVE ranged from 0.504 to 0.664, and all were above the recommended value of 0.5 (Fornell and Larcker, 1981). This indicated that the model in this study showed good convergent validity. Then, we further assessed the discriminant validity of the model. According to the results in Table 4, the square roots of the AVEs for all constructs were greater than the corresponding correlations between the constructs, which indicated that the present measurement model had good discriminant validity (Fornell and Larcker, 1981).

**TABLE 3:** Results of confirmatory factor analysis.

Construct	Items	Loading	Cronbach’s α	AVE	CR
Reappraisal	6	0.568	0.856	0.510	0.860
		0.663			
		0.638			
		0.820			
		0.772			
		0.790			
Suppression	4	0.689	0.801	0.504	0.803
		0.713			
		0.722			
		0.716			
Guanxi harmony	3	0.689	0.811	0.560	0.817
		0.820			
		0.807			
Employee Motivation	4	0.727	0.883	0.664	0.887
		0.793			
		0.897			
		0.832			

SD, standard deviation; CR, composite reliability; AVE, average variance extracted.

**TABLE 4:** Means, standard deviations and correlations.

Variables	Mean	SD	Reappraisal	Suppression	Guanxi harmony	Employee motivation	Gender	Age	Income/year
Reappraisal	5.528	0.813	0.714						
Suppression	3.689	1.185	−0.050	0.710					
Guanxi harmony	3.710	0.770	0.308**	−0.133**	0.748				
Employee motivation	4.141	0.755	0.367***	−0.122**	0.503***	0.815			
Gender	NA	NA	0.055	−0.238**	−0.116*	0.060	NA		
Age	NA	NA	0.067	0.113*	0.143**	0.103*	−0.165***	NA	
Income/year	NA	NA	0.005	−0.088	0.058	0.034	−0.012	0.078	NA

NA, not applicable. The square roots of AVE are the numbers in the diagonal row. *p < 0.05, **p < 0.01, ***p < 0.001.

Additionally, the results of model fit values in Table 2 showed that our four-factor measurement model had a good model fit. The Chi-square by degree of freedom (χ2/df) = 2.492, comparative fit index (CFI) = 0.955, Tucker-Lewis index (TLI) = 0.945, root mean square error of approximation (RMSEA) = 0.055, and standardized root mean square residual (SRMR) = 0.046 were all met the recommended level (Hair et al., 2013).

### Correlation analysis

The means, standard deviations, and correlations of the variables were given in Table 4. Table 4 showed that reappraisal was significantly positively correlated with guanxi harmony (*r* = 0.308, *p* < 0.001) and employee motivation (*r* = 0.367, *p* < 0.001). Suppression was significantly negatively correlated with guanxi harmony (*r* = 0.133, *p* < 0.01) and employee motivation (*r* = 0.122, *p* < 0.01); and guanxi harmony was significantly positively correlated with employee motivation (*r* = 0.503, *p* < 0.001). This provided a preliminary verification for the hypotheses testing that followed.

### Test of hypotheses

#### Test of main effects

We used hierarchical regression through SPSS 24.0 to analyze the influential relationships between reappraisal, suppression and guanxi harmony, and employee motivation, and the results of the analysis are shown in Table 5. We first tested the effect of emotion regulation strategies on employee motivation. After controlling for the demographic variables of gender, age, and annual income, we used employee motivation as the dependent variable, and we added two dimensions of the independent variable emotion regulation, reappraisal and suppression, to Model 6 and Model 8, respectively. According to the results in Table 5, reappraisal had a significant positive effect on employee motivation (Model 6, β = 0.341, *p* < 0.001) and suppression had a significant negative effect on employee motivation (Model 8, β = −0.097, *p* < 0.01). Therefore, H1 and H2 were supported.

**TABLE 5:** Results of hierarchical regression analysis.

Variable	Guanxi harmony				Employee motivation					
	Model 1	Model 2	Model 3	Model 4	Model 5	Model 6	Model 7	Model 8	Model 9	Model 10
Gender	−0.147*	−0.180**	−0.210**	−0.237**	–0.067	–0.105	–0.030	–0.119	–0.019	–0.055
Age	0.032**	0.026*	0.036**	0.029**	0.024*	0.016	0.006	0.027*	0.010	0.007
Income/year	0.034	0.034	0.021	0.022	0.018	0.018	0.004	0.008	–0.002	0.000
Reappraisal		0.292***		0.285***		0.341***	0.219***			0.220***
Suppression			−0.116***	−0.107***				−0.097**	–0.041	–0.043
Guanxi harmony							0.416***		0.478***	0.405***
F	5.268**	17.362***	7.881***	17.125***	2.149	20.757***	42.251***	4.339**	33.635***	35.820***
*R* ^2^	0.032	0.125	0.061	0.151	0.013	0.146	0.304	0.035	0.258	0.295
△*R*^2^		0.093	–0.064	0.090		0.133	0.158	–0.269	0.223	0.037

*p < 0.05, **p < 0.01, ***p < 0.001.

#### Test of mediation

To examine the mediating role of guanxi harmony among reappraisal, suppression, and employee motivation, we added reappraisal and guanxi harmony to Model 7, and suppression and guanxi harmony to Model 9. As shown in Model 7, it can be seen that guanxi harmony had a significant positive effect on employee motivation (β = 0.416, *p* < 0.001), and the association between reappraisal and employee motivation was still significant (β = 0.219, *p* < 0.001), but the coefficient decreased from 0.341 to 0.219, indicating that guanxi harmony partially mediated the relationship between reappraisal and employee motivation, and H3 was supported. Similarly, as shown by Model 9, guanxi harmony had a significant positive effect on employee motivation (β = 0.478, *p* < 0.001), but the association between suppression and employee motivation was no longer significant (β = −0.041, *p* > 0.05), indicating that guanxi harmony fully mediated the association between suppression and employee motivation, and H4 was supported.

To further test the indirect association between emotion regulation strategies, guanxi harmony and employee motivation, mediation analysis using PROCESS macro (model 4) was conducted with 5,000 bootstrapped resamples, following the procedures recommended by Hayes (Hayes et al., 2017). The results showed that guanxi harmony had a significant mediating effect in the relationship between reappraisal [β = 0.164, 95% bias-corrected bootstrapped confidence interval (CI) = 0.078, 0.176], and suppression (β = −0.042, 95% bias-corrected bootstrapped CI = −0.077, −0.011) and employee motivation, further supporting H3 and H4. The mediating effect was verified as the values of CI around the indirect effect did not contain zero.

## Discussion

Our results confirmed all the hypotheses. First, we verified that different types of emotion regulation strategies affect employee motivation differently. We found that suppression is still detrimental to employee motivation even in the Chinese context, whereas reappraisal can improve employee motivation. That is, although suppression can have some benefits for emotion regulation in the Chinese culture (Yeung and Fung, 2012), it is detrimental to employee motivation. However, it is noteworthy that although suppression had a negative effect on employee motivation, its intensity was lower than reappraisal, which might indicate that although suppression was not conducive to employee motivation, its damage was not so severe. This may be related to the unique cultural context of the East where the widespread use of suppression and its many benefits have muted the detrimental effects of this strategy on employee motivation.

Second, we verified the mediating role of guanxi harmony in the relationship between emotion regulation strategies and employee motivation. We found that suppression impaired employee motivation by damaging interpersonal relationships, whereas reappraisal promoted employee motivation by facilitating interpersonal relationships. This validated the importance of interpersonal coordination in Chinese culture. Although Western countries emphasize promoting leadership through institutions and other aspects of laws, the human factor of relationships plays an important role in human resource management in Chinese culture. It implied that leaders need to draw on the important power of interpersonal relationships if they tend to influence employee motivation through emotions. And although in many perceptions, individuals in Eastern cultures tend to use suppression to maintain interpersonal relationships, the results of this study found that this tendency was not always beneficial to interpersonal coordination and in fact suppression was detrimental to interpersonal relationships, which is similar to the conclusions approved in Western countries (Zhao and Zhao, 2015). Fortunately, the intensity with which suppression damaged interpersonal relationships and the intensity with which the interpersonal relationships further damaged employee motivation is not so severe (Wei et al., 2013). Therefore, the differences between different cultures regarding the influence of emotion regulation strategies on employee motivation may lie in the type and intensity of the emotion regulation strategies applied (Ding et al., 2017).

### Theoretical implications

First, the EASI model emphasized the importance of emotion on motivation, but little research has been done from the perspective of emotion regulation. It is usual for leaders to have emotional fluctuations in the face of challenging environments, the key to keeping the work effective is how to reduce the negative impact of emotions. We integrated the EASI model with COR theory to explore the different effects of two emotion regulation strategies (i.e., reappraisal and suppression) on employee motivation. By introducing emotion regulation strategies from proactive and interventional perspectives, we can expand the applicability of the theory and provide a theoretical path for using emotion regulation strategies to increase employee motivation.

Second, previous studies on the emotion-related processes of leaders influencing employees have mainly been conducted from a psychological perspective (Gable and Harmon-Jones, 2010). Recently, attention has been paid to the social factor of interpersonal emotion regulation of leaders in Western contexts, which provides a new path to understanding how leaders influence followers (Vasquez et al., 2021). The present study then supplemented this affective influence process with evidence from Eastern contexts. This study introduced the important interpersonal concept of guanxi harmony, which is emphasized in traditional Chinese and oriental cultures, and verified the important mediating role of guanxi in the process of different types of emotion regulation strategies affecting employee motivation. This study further validates the important role of guanxi in the Chinese business environment and expands its role as an important mediator in the cross-level influence of leaders on employees, especially in the process of affective influence, which enriches the studies on the mechanisms of guanxi in the influence of leaders on employees in the Eastern cultural context.

Third, our findings appear to support a generational shift in the outcomes of Chinese people’s use of emotion regulation strategies. Previous studies have shown that traditional Chinese culture not only encourages suppression as an emotion regulation strategy, but also can benefit from such strategy (Yuan et al., 2015; Zhou et al., 2016), even in terms of interpersonal harmony (Wei et al., 2013), which differs significantly from the negative attitudes toward suppression and its negative influential outcomes of Western cultures (Soto et al., 2011; Nam et al., 2018). However, a growing body of recent research on suppression and social functions has found consistency between Chinese and Western findings that Chinese youth may have more difficulty benefiting from suppression strategies (Kwon and Kim, 2019; Yeung and Wong, 2020). Consistently, the results of the present study show a negative effect of suppression on guanxi and employee motivation, as well as a negative correlation between suppression and age, supporting the idea that Chinese young adults are developing cross-cultural consistency with Westerners in emotion regulation strategies.

### Management implications

First, we found that although leaders in Eastern cultures tended to apply a suppression strategy at work to reduce the impact of negative emotions, in reality, such strategy is detrimental to employee motivation and this damage is achieved by damaging interpersonal relationships. That is, even though the suppression strategy can have some benefits in Eastern cultures (Soto et al., 2011; Nam et al., 2018), it harms interpersonal coordination of collective and employee motivation. Therefore, leaders should avoid using the suppression strategy when facing negative or motivation-required situations.

Second, in contrast to the unrecommended use of the suppression strategy, we found that reappraisal is an effective strategy that can lead to enhanced interpersonal relationships and employee motivation. Based on the different effects of different emotion regulation strategies on employee motivation, appropriate emotion regulation training should be included in corporate practices to enhance leadership, and leaders should be trained and encouraged to use appropriate emotion regulation strategies proactively (e.g., reappraisal) in situations that require motivation.

Finally, the findings of this study further demonstrate the importance of interpersonal relationships in human resource management in the Chinese context. That is, well-coordinated interpersonal relationships promote motivation but poor interpersonal relationships undermine motivation. Although the importance of institutional factors is emphasized in organizations, the important power of human factors cannot be ignored in the Chinese context (Hmieleski and Baron, 2009; Li et al., 2019). Therefore, in the management activities, leaders should pay more attention to the cultivation and coordination of interpersonal relationships in order to facilitate employee motivation and promote work efficiency.

## Conclusion

We found that different emotion regulation strategies of leaders can have opposite direct effects on employee motivation. Moreover, guanxi harmony, a culturally specific interpersonal phenomenon in China, mediates this effect of emotion regulation strategies and employee motivation. Our findings expand the study of leadership effectiveness from the perspective of emotion regulation and interpersonal relationships, and provide constructive suggestions for leaders to improve leadership effectiveness in organizations.

## Data availability statement

The raw data supporting the conclusions of this article will be made available by the authors, without undue reservation.

## Ethics statement

The studies involving human participants were reviewed and approved by Institutional Review Board of the School of Business (No. AHUT 20210701 001). The patients/participants provided their written informed consent to participate in this study.

## Author contributions

WZ and LC designed the concept of the manuscript, collected data, wrote and revised the manuscript, and finally approved the version to be published. YH edited the language of the manuscript. SG analyzed and interpreted the data. WZ, SG, and YH co-drafted the manuscript. JL and MS supervised the data gathering process. All authors contributed to the article and approved the submitted version.
